# Seaweed Extract-Stimulated Priming in *Arabidopsis thaliana* and *Solanum lycopersicum*

**DOI:** 10.3390/plants10112476

**Published:** 2021-11-16

**Authors:** Md Tohidul Islam, Tony Arioli, David M. Cahill

**Affiliations:** 1 School of Life and Environmental Sciences, Deakin University Geelong Waurn Ponds Campus, Waurn Ponds, VIC 3216, Australia; md.islam2@deakin.edu.au (M.T.I.); tonyarioli@seasol.com.au (T.A.); 2 Seasol International, Bayswater, VIC 3153, Australia

**Keywords:** seaweed, *Ascophyllum nodosum*, *Durvillaea potatorum*, *Phytophthora cinnamomi*, *Arabidopsis thaliana*, priming, priming phase, post-challenge primed state

## Abstract

Plant priming is an induced physiological state where plants are protected from biotic and abiotic stresses. Whether seaweed extracts promote priming is largely unknown as is the mechanism by which priming may occur. In this study, we examined the effect of a seaweed extract (SWE) on two distinct stages of plant priming (priming phase and post-challenge primed state) by characterising (i) plant gene expression responses using qRT-PCR and (ii) signal transduction responses by evaluating reactive oxygen species (ROS) production. The SWE is made from the brown algae *Ascophyllum nodosum* and *Durvillaea potatorum.* The priming phase was examined using both *Arabidopsis thaliana* and *Solanum lycopersicum*. At this stage, the SWE up-regulated key priming-related genes, such as those related to systemic acquired resistance (SAR) and activated the production of ROS. These responses were found to be temporal (lasting 3 days). The post-challenge primed state was examined using *A. thaliana* challenged with a root pathogen. Similarly, defence response-related genes, such as *PR1* and *NPR1*, were up-regulated and ROS production was activated (lasting 5 days). This study found that SWE induces plant priming-like responses by (i) up-regulating genes associated with plant defence responses and (ii) increasing production of ROS associated with signalling responses.

## 1. Introduction

Agricultural biostimulants made from seaweed extracts have received considerable attention in recent years due to their use in conventional, sustainable, and regenerative agriculture. Seaweed extracts have been demonstrated to increase crop productivity, increase nutrient use, and enhance plant tolerance to biotic and abiotic stress [1]. Extracts from a single macroalgae, such as *Ascophyllum nodosum*, can stimulate an increase in plant growth, and increase crop productivity [2,3,4]. Other extracts derived from two brown algal species, *A. nodosum* and *Durvillaea potatorum*, also stimulated tomato plant growth and productivity and improved soil health [5].

Seaweed extracts are compositionally diverse and complex in nature [3]. Recent reviews [6,7] confirm the great diversity in extracts derived from macroalgae and the wide variety of physiological responses to specific components of the extracts. For example, Ghaderiardakani et al. [8] found that extracts from Ulva species contained a range of hormones that had both inhibitory and stimulatory effects on plant growth and development. It is well recognised that extracts from various major groups of macroalgae, including the two species used in the current study, contain a wide range of biologically active compounds including plant hormones such as cytokinins [9], laminarins, alginates, phenolics, ulvans, and carrageenans [6,10,11]. However, the mechanisms that underly the effect of seaweed extracts on plants remains unclear. Also, it is unknown if extracts made from one or two macroalgae use the same stimuli.

In our previous manuscript [12], we compared the early-stage defence responses (up to 24 h) in *A. thaliana* plants infected with *P. cinnamomi* following the application of three different types of seaweed extracts. In this study, we hypothesised that the mechanism of action of seaweed extracts involves a plant priming-like response. Here, we have used a seaweed extract (derived from two brown algal species, *A. nodosum* and *D. potatorum*) to compare plant responses at two distinct plant priming stages (non-stressed and stressed) to characterise the priming responses, timing patterns, and response durations over a time course up to 5 days.

The factors that initiate priming in plants are referred to as “priming stimuli” [13]. The priming phenomenon consists of three stages: the priming phase (unstressed), the post-challenge primed state (stressed) and the transgenerational primed state. The priming phase may be transient or maintained throughout the lifetime of the plant and can potentially be inherited by subsequent generations [13,14,15]; research on several species has demonstrated that this induction may last for multiple generations [16]. Different priming stimuli may result in similar priming-related changes or changes that are specific to a particular interaction [13]. During the priming phase, only slight alterations in primary and secondary metabolism appear to be required for plants to be in a standby state of alertness [17].

At the post-challenge primed state, plants show an increased activation of plant defence responses against pathogen attack. For example, *Arabidopsis thaliana* plants treated with a range of priming stimuli were found to have increased resistance to a virulent strain of *Pseudomonas syringae* through the primed accumulation of ROS, SA, and pathogenesis-related protein 1 (PR1) [18]. Additionally, the SA receptor, the nonexpressor of pathogenesis-related gene 1 (NPR1), was identified as a positive regulator in SA-induced priming in *A. thaliana* against *P. syringae* [19,20]. However, whether SWE induce defence-related signalling pathways at the post-challenge primed state that acts against a root pathogen needs to be determined.

Therefore, the aims of the present study were to determine (i) if a plant priming-like response was induced by a seaweed extract derived from the two species, *A. nodosum* and *D. potatorum*, in *A. thaliana*; (ii) if the seaweed extract-induced priming-like response in *A. thaliana* is conserved across species; and (iii) the duration of any priming-like response and how that may influence the interaction between *A. thaliana* and a root pathogen.

We show that the seaweed extract-induced responses (i) are temporal in nature, (ii) involve the activation and production of ROS, (iii) enhance the expression of major priming-related genes, and (iv) collectively indicate that this SWE induces plant priming.

## 2. Results

### 2.1. Gene Expression in Arabidopsis thaliana and Solanum lycopersicum Following One Application of SWE at the Plant Priming Phase

#### 2.1.1. Priming-Related Gene Expression in *A. thaliana*

**Figure 1:**
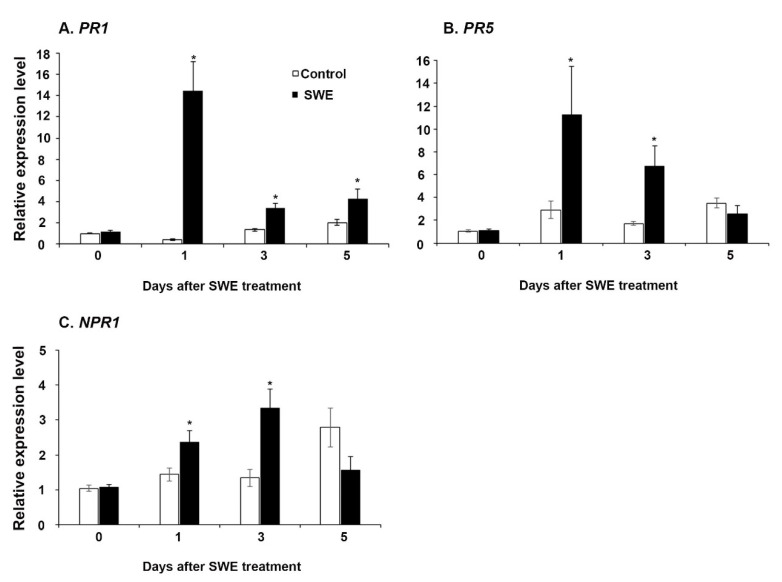
Relative quantification of the expression (fold change) of priming-related genes: (**A**) *PR1*, (**B**) *NPR1*, and (**C**) *PR5* in *A. thaliana* at 0, 1, 3, and 5 days after a single application of SWE or water as the control. Data shown are the mean of three independent biological replicates (each replicate consisted of 10 plants) and bars represent the standard error of the mean. * denotes significant difference (*p* = 0.05) between SWE and control samples at each time point according to Duncan’s multiple range test.

The four time points at 0, 1, 3, and 5 days after the single SWE application were examined using RT-qPCR for priming-related gene expression. The key priming-related genes, pathogenesis-related protein 1 *(PR1)*, pathogenesis-related protein 5 *(PR5)*, and non-expressor of pathogenesis protein 1 *(NPR1)* were selected to investigate their expression in this study [21]. Each gene showed variable expression across the time points following prior treatment of plants with the extracts. Among the three genes, *PR1* was consistently significantly up-regulated at all time points compared to the respective water control (Figure 1A). Both *NPR1* and *PR5* were significantly up-regulated at 1 day and 3 days and then showed reduced expression at 5 days after treatment (Figure 1B,C).

#### 2.1.2. Key Defence Priming-Related Gene Expression in *A. thaliana*

**Figure 2:**
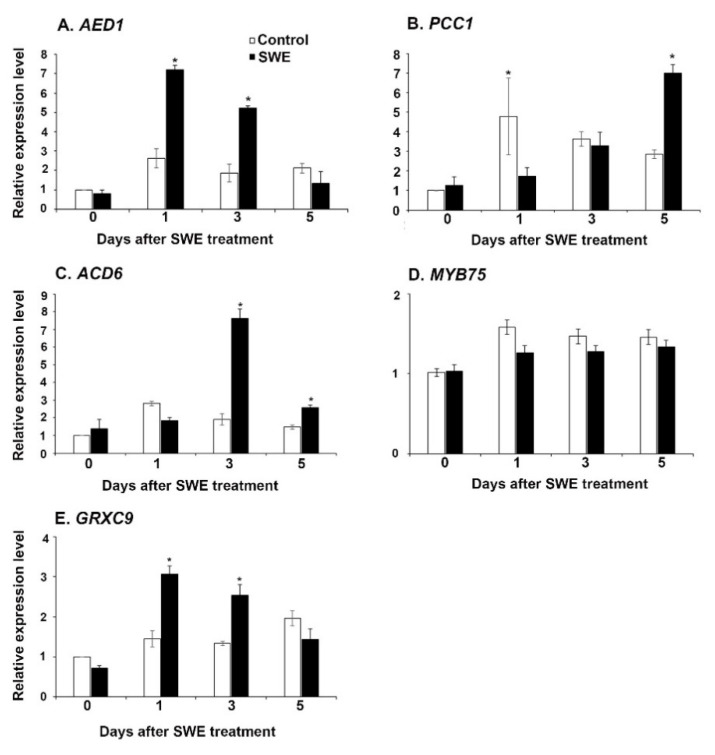
Relative quantification of the expression (fold change) of defence priming-related genes: (**A**) *AED1,* (**B**) *PCC1* (**C**) *ACD6*, (**D**) *MYB75*, and (**E**) *GRXC9* in *A. thaliana* following a single application of SWE or water as the control. Data shown are the mean of three independent biological replicates (each replicate consisted of 10 plants) and bars represent the standard error of the mean. * denotes significant difference (*p* = 0.05) between SWE and control samples at each time point according to Duncan’s multiple range test.

Based on their known involvement in plant defence pathways, the expression of *apoplastic enhanced disease susceptibility-dependent 1 (AED1)* [22], *pathogen and circadian controlled 1(PCC1)* [23], *accelerated cell death 6 (ACD6)* [24], *glutaredoxin-C9 (GRXC9)* [25], and the transcription factor *MYB75* [26], was also investigated. The relative expression levels were determined at 0, 1, 3, and 5 days after SWE application. The expression levels of *AED1* and *GRXC9* were significantly up-regulated at 1 and 3 days after SWE treatment (Figure 2A,E), whereas the expression of *PCC1* was significantly up-regulated at 5 days in SWE treatment compared to the respective control (Figure 2B). Moreover, the expression of *ACD6* was significantly up-regulated at 3 and 5 days after SWE treatment (Figure 2C). However, the expression level of *MYB75* was not significantly different than that of the respective controls (Figure 2D).

#### 2.1.3. ROS-Associated Gene Expression in *A. thaliana*

**Figure 3:**
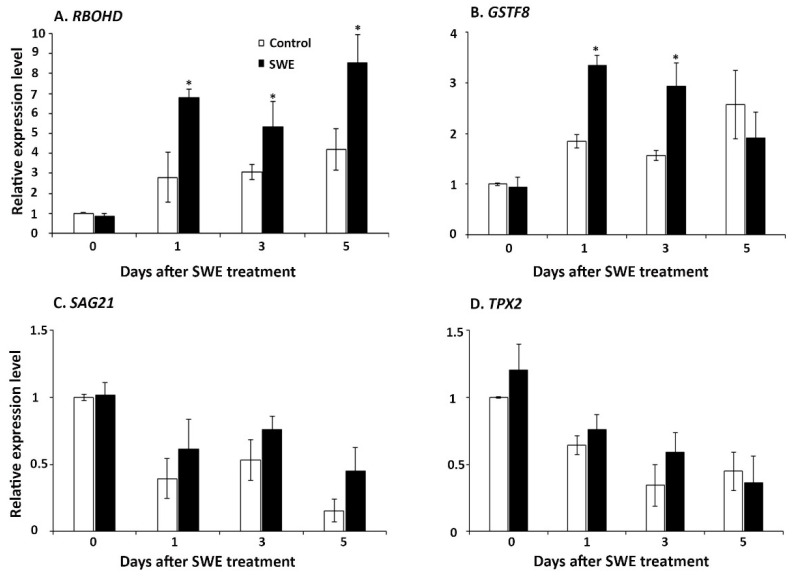
Relative quantification of the expression of ROS-associated genes: (**A**) *RBOHD*, (**B**) *GSTF8,* (**C**) *SAG21*, and (**D**) *TPX2* at 0, 1, 3, and 5 days after a single application of SWE or water as the control. Data shown are the mean of three independent biological replicates (each replicate consisted of 10 plants) and bars represent the standard error of the mean. * denotes significant difference (*p* = 0.05) between SWE and control samples at each time point according to Duncan’s multiple range test.

The three time points assessed after the single seaweed extract application were also examined for ROS-related gene expression. Four genes were examined: (1) *respiratory burst oxidase protein D* *(RBOHD)*, which has multiple roles in controlling cell death [27]; (2) *glutathione S-transferase Phi8 (GSTF8)*, which is involved in detoxification of ROS [28]; (3) *senescence-associated gene 21 (SAG21)*, which mediates tolerance to oxidative stresses [29]; and (4) *targeting protein for XKLP2* (*TPX2*), which is involved in cellular responses to oxidative stress [30]. These key ROS-related genes showed varying patterns of expression at each time point after SWE application. Among the four genes, *RBOHD* was significantly up-regulated at 1, 3, and 5 days compared to the respective control, whereas *GSTF8* was significantly up-regulated at only 1 and 3 days after SWE application (Figure 3A,B). However, the expressions of the other two ROS-associated genes (*SAG21* and *TPX2*) were not significantly higher at all tested time points compared with their respective controls (Figure 3C,D).

#### 2.1.4. Priming-Related Gene Expression in *S. lycopersicum*

**Figure 4:**
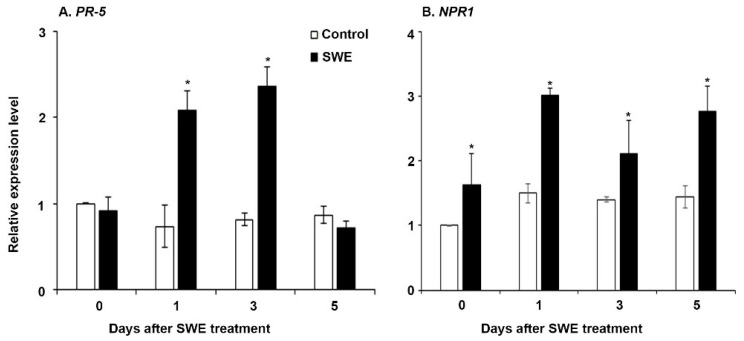
Relative quantification of the expression (fold change) of priming-related genes: (**A**) *PR5* and (**B**) *NPR1* in *S. lycopersicum* at 0, 1, 3, and 5 days after a single application of SWE or water as the control. Data shown are the mean of three independent biological replicates (each replicate consisted of 10 plants) and bars represent the standard error of mean. * denotes significant difference (*p* = 0.05) between SWE and control samples at each time point according to Duncan’s multiple range test.

The two key-priming-related genes (*PR5* and *NPR1*) [31] were selected for examination of their expression in *S. lycopersicum* at the three time points after a single SWE application. The expression levels for both genes were significantly higher across the time points in the treated plants compared to those of the respective water controls (Figure 4). However, *PR5* expression was not significantly higher at 5 days after application.

#### 2.1.5. ROS Production Changes in Response to Treatment with SWE on the Plant Priming Phase in *Arabidopsis thaliana* and *Solanum lycopersicum* Following One Application of SWE

##### Detection and Quantification of ROS Related Responses in *A. thaliana*

**Figure 5:**
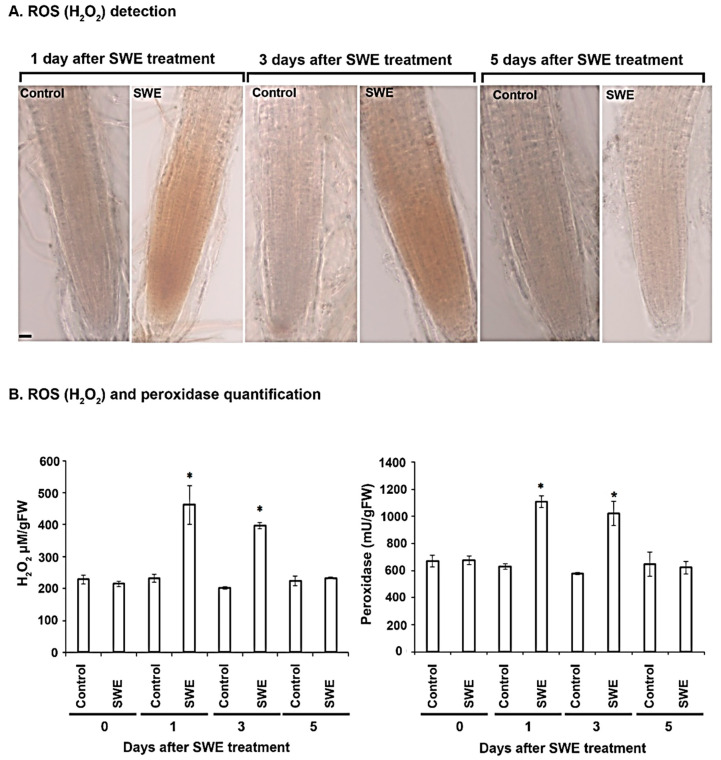
ROS and peroxidase quantification in *A. thaliana* roots following a single application of SWE or water as the control. Hydrogen peroxide (H_2_O_2_) was detected with 3,3′-diaminobenzidine (DAB) stain, resulting in a reddish-brown precipitate in the root tissue. (**A**). The production of H_2_O_2_ was detected in roots that were examined after 1 and 3 days following a single application of SWE. No H_2_O_2_ production was found in roots examined 5 days after SWE treatment or in all controls. Scale = 20 µm. (**B**) Quantification of H_2_O_2_ and peroxidase showed that there was a significantly high concentration of each component at 1 and 3 days after the SWE application compared to the respective controls. Data shown are the mean of three independent biological replicates (each replicate consisted of 10 plants) and bars represent the standard error of the mean. * denotes a significant difference (*p* < 0.05) in the treated compared to each control according to Duncan’s multiple range test.

The production of H_2_O_2_ was investigated as a potential component involved in SWE-induced priming. The presence of a reddish-brown precipitate in roots following staining with DAB was used as a measure of H_2_O_2_ accumulation. At one day after a single application of SWE, H_2_O_2_ was increased in the roots in comparison with the controls (Figure 5A). A similar result was found at three days after SWE treatment. No H_2_O_2_ was detected in treated roots after 5 days.

Peroxidase activity was also measured in *A. thaliana* seedlings treated with a single application of SWE. On the day of treatment, the levels of H_2_O_2_ in the treated and control roots were the same. A significant difference in H_2_O_2_ concentration was found in the roots of *A. thaliana* at 1 day after application compared to the respective control. A similar difference was also found at 3 days after the treatment, but at 5 days after treatment the levels of H_2_O_2_ were the same in treated and control roots. For peroxidase, a similar trend of heightened levels at 1 and 3 days after treatment was found (Figure 5B).

##### Detection and Quantification of ROS Related Responses in *S. lycopersicum*

**Figure 6:**
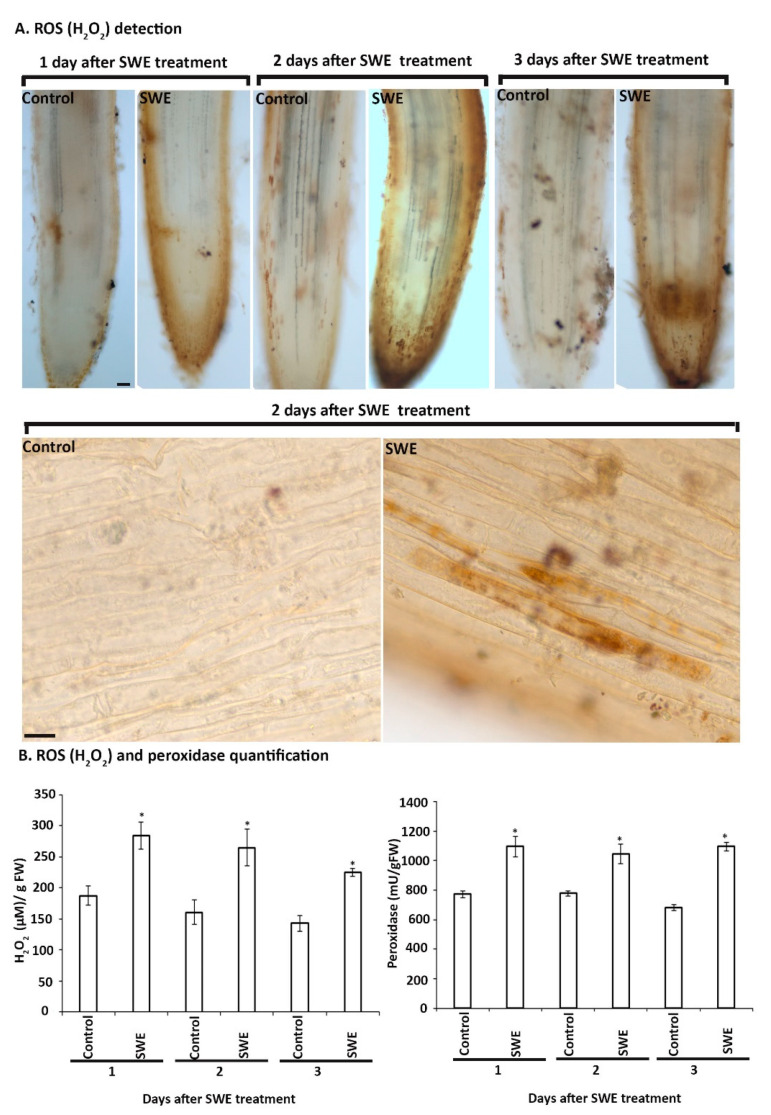
Detection and quantitation of hydrogen peroxide and peroxidase in *S. lycopersicum* roots grown in the sand culture system and treated with a single application of SWE or water as the control. (**A**). The SWE-treated roots harvested at 1, 2 and 3 days after the application showed higher production of H_2_O_2_ compared to their respective control. Scale bar = 50 µm. Individual root cells 2 days after SWE application were DAB positive. Scale bar = 20 µm (**B**). H_2_O_2_ and peroxidase quantification from tomato roots grown with SWE or water as the control. Data shown are the mean of three independent biological replicates (each replicate consisted of 10 plants) and bars represent the standard error of the mean. * denotes the significant difference in the treatment compared to the respective control at *p* < 0.05 according to Duncan’s multiple range test.

Based on the findings of SWE treatment of *A. thaliana*, we examined the production of H_2_O_2_ and peroxidase over a shorter time course, that is, up to three days following a single treatment of *S. lycopersicum* roots with SWE. At each time point after treatment, H_2_O_2_ was detected in the roots (Figure 6A). No H_2_O_2_ or only low basal levels were detected in the control roots. The production of H_2_O_2_ in roots was found to be associated with either single cells or groups of cells, especially at 2 and 3 days after SWE treatment (Figure 6A and Supplementary Figure S3). In addition, the quantification of H_2_O_2_ and peroxidase levels showed a significantly higher amount of both at all days after SWE treatment compared with their respective controls (Figure 6B).

### 2.2. Gene Expression in A. thaliana at the Post-Challenge Primed State after Two Treatments with SWE and Then Inoculation with Phytophthora cinnamomi

#### 2.2.1. Defence Priming-Related Gene Expression in the Post-Challenge Primed State

**Figure 7:**
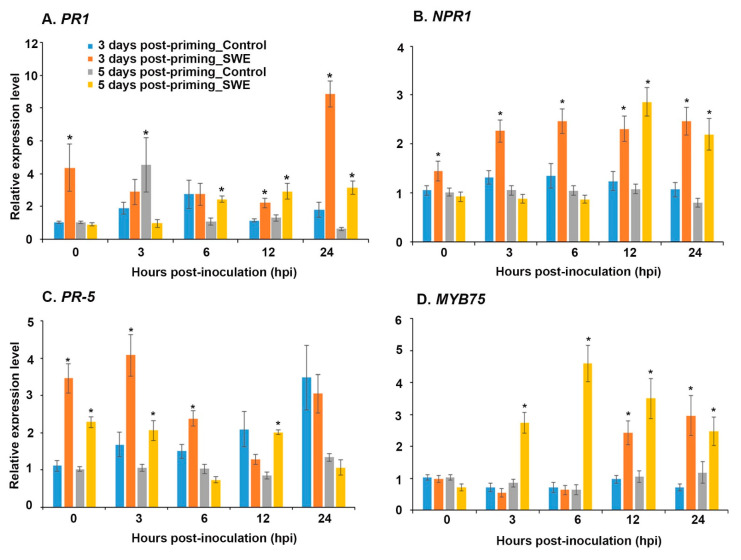
Relative quantification of the expression of defence priming-related genes: (**A**) *PR1*, (**B**) *NPR1,* (**C**) *PR5*, and (**D**) *MYB75* at 0, 3, 6, 12, and 24 h post-inoculation (hpi) at 3 and 5 days post-priming. Data shown are the mean of three independent biological replicates (each replicate consisted of 10 plants) and bars represent the standard error of the mean. * denotes the significant difference (*p* = 0.05) between SWE and control samples at each time point according to Duncan’s multiple range test.

The expression level of key defence-priming-related genes was examined in *A. thaliana* at 3 and 5 days after a second treatment with SWE and following inoculation with *P. cinnamomi* (Figure 7). At 3 days post-priming and at the time of inoculation (0 h), *PR1*, *NPR1*, and *PR5* showed significant up-regulation of expression compared to the water-treated control. It is worth noting that even though the expression level of *NPR1* was considerably lower than that of *PR1* and *PR5*, the level of expression was maintained over the 24 h time period (Figure 7A–C). *PR5* showed diminishing expression over the same time period from a relatively high level at 3 hpi (Figure 7C). In contrast, *PR1* showed a similar expression level at 3 and 6 h post-inoculation and then up-regulation in expression from 12 h onwards compared to the respective control (Figure 7A). Similarly to the expression of *PR1*, *MYB75* showed significantly increased expression from 12 hpi onwards but showed similar or reduced expression from 0 to 6 hrs after inoculation (Figure 7D)

At 5 days post-priming, the expression levels of *PR1*, *NPR1*, and *MYB75* were not statistically different at 0 hpi compared to the control; however, they were found to be significantly up-regulated at later time points following inoculation (Figure 7A,B,D). Even though *PR5* showed a statistically similar expression level at 6 and 24 hpi, the expression level was significantly up-regulated at 0, 3, and 12 hpi (Figure 7C).

#### 2.2.2. ROS Production during the Post-Challenge Primed State in *A. thaliana* Following Two Treatments with SWE and then Inoculation with *Phytophthora cinnamomi*

##### ROS Detection at the Post-Challenge Primed State

**Figure 8:**
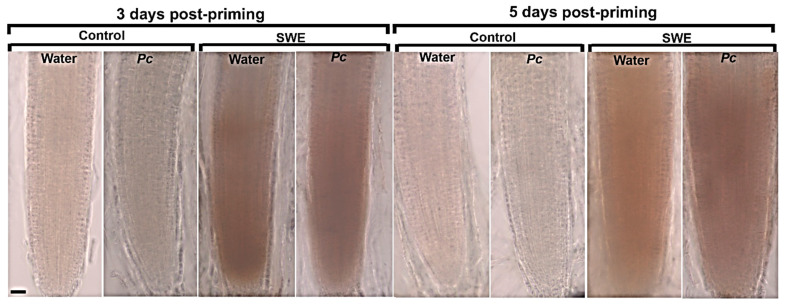
Hydrogen peroxide detection in *A. thaliana* roots at 3 and 5 days post-priming. Plants were exposed to two applications of SWE, or water as the control, followed by inoculation with *Phytophthora cinnamomi* (*Pc*) zoospores or mock inoculation with water. All plants were grown for 7 days in sand and were then harvested at 12 h post-inoculation. Control roots showed no H_2_O_2_ production, but SWE-treated roots showed H_2_O_2_ production. Scale bar = 20 µm. Images are representative of three independent biological replicates each with at least 12 roots.

At 3 days post-priming, H_2_O_2_ was not detected in control roots (Figure 8). *A. thaliana* ecotype Ler is susceptible to *P. cinnamomi* [32] and therefore, we would not expect ROS to be produced following infection. However, H_2_O_2_ was detected in roots treated with SWE alone and in roots treated with SWE and then inoculated with *P. cinnamomi*. A very similar result was found in roots at 5 days post-priming, that is, increased H_2_O_2_ production in those roots treated with SWE alone or treated with SWE and then inoculated.

## 3. Discussion

This research provides new insights into how seaweed extracts prime plants that results in individual plant and agricultural benefits. We have shown that a brown algal extract, made from *A. nodosum* and *D. potatorum*, is able to stimulate the plant priming mechanism in both *A. thaliana* and *S. lycopersicum*. The plant priming mechanism is relevant to agriculture because it underpins the behaviour of plants to enable them to tolerate and adapt to stresses that are encountered during growth.

We have now undertaken, for the first time, a comprehensive study that has incorporated different application regimes and then analysis across successive time points with and without the imposition of a biotic stress to explore the process of priming that is induced by SWE treatment at both the initial priming phase and, in detail, at a post-challenge primed state. We used *P. cinnamomi* as a representative stress to challenge plants after treatment with the seaweed extract and to synchronise the timing for the post-challenge stress event. Our use of a well characterised model plant system and then extension into a common horticultural species has proven to be a powerful approach for elucidating the molecular priming responses induced by a biostimulant. It is evident from a recent study [5] that a seaweed extract made from *A. nodosum* and *D. potatorum* enhanced *S. lycopersicum* growth and productivity; likewise, in our laboratory system we confirmed a strong positive effect of SWE on health and root growth (Supplementary Figure S4). Priming in our study has been found to be strongly correlated with the expression of key priming-related genes and production of the reactive-oxygen species, H_2_O_2_, across both priming phases. Even though in this study we have not directly measured the impact of SWE treatment on pathogen growth, the measured changes in defence-associated gene expression and ROS production strongly suggest that the primed plants are more resistant to infection.

### 3.1. Effect of SWE on the Priming Phase in A. thaliana and S. lycopersicum

Priming enhances the defence capacity of plants and priming agents act to initiate and activate defence mechanisms [13]. Defence priming is well documented in several studies of PR-protein accumulation and enzymatic activity; for example, the expression levels of *PR1*, *PR2*, and *PR5* were much higher in beta-aminobutyric acid (BABA)-treated *A. thaliana* plants when exposed to a bacterial pathogen [33]. Moreover, NPR1 is considered an essential regulator that is required for long-lasting priming against biotic stress. For example, *Pseudomonas putida* LSW17S elicits protection against several pathogens in various plant species and it was revealed that *P. putida* LSW17S-induced priming in *A. thaliana* partly depends upon NPR1-dependent disease resistance [21]. In our previous study, we had shown the involvement of these genes in seaweed extract-induced priming at the post-challenge primed state in *A. thaliana* following continuous application of the extracts prior to inoculation [12]. Now, in the current study, we have shown that the priming response at the priming phase can be stimulated using only a single application of the same seaweed extract. Gene expression analysis has now also shown that both *A. thaliana* and *S. lycopersicum* activate the expression of major priming-related genes at the priming phase. Importantly, a heightened expression of these genes was apparent for three days after application of SWE, demonstrating the activation of priming by SWE, and the maintenance of the priming phase for at least three days.

To further explore the priming response following SWE treatment, we also investigated the expression of five defence-related genes (*AED1*, *PCC1*, *ACD6*, *MYB75*, and *GRXC9*) in *A. thaliana* following a single application of SWE. All of these genes were up-regulated in their expression at least at one time point up until 5 days after SWE application. *AED1* encodes a predicted aspartyl protease that has been reported to be induced locally and systematically during SAR signalling and locally by salicylic acid (SA) [22]. The other three defence-related genes (*PCC1*, *ACD6*, and *GRXC9*) are associated with SA-induced plant defence pathways [23,24,25,34]. For example, *GRXC9*, which encodes a CC-type glutaredoxin from *A. thaliana*, is an SA-responsive gene induced early and transiently by an NPR1-independent defence pathway [25,35]. Also, *MYB75*, which encodes transcription factor MYB75 studied here, is a positive regulator of the production of anthocyanin, a secondary metabolite that defends from invasion by pathogens [26]. Our study has shown the up-regulation of *NPR1* at 1 and 3 days after SWE application; therefore, the up-regulation of *GRXC9* at the same time indicates that this gene likely acts on an NPR1-dependent pathway in SWE-induced priming. Therefore, the induction of all four SA-associated genes strongly indicates that SA signalling pathways are associated with SWE-induced priming in *A. thaliana.*

The association of reactive oxygen species (ROS) with priming has been reported following treatment of various plant species with priming agents such as β-aminobutyric acid (BABA) [36]. For example, ROS was produced in BABA-treated grapevine leaves in response to the downy mildew pathogen *Plasmopara viticola* [37]. In the current study, ROS production was demonstrated in the absence of a pathogen in both *A thaliana* and *S. lycopersicum* up to three days after the plants were exposed to a single application of SWE. ROS is a major redox (reduction–oxidation reaction) metabolite and it induces cellular oxidative damage at high concentrations, which can cause cell death [38]. Plant peroxidases contribute to ROS scavenging by their peroxidative (or catalytic) activity, and they can also generate superoxide radicals (O_2_^−^) via their oxidative cycle. The oxidative cycle is involved in the building up of high levels of ROS during the oxidative burst [39,40]. It is notable that the initial peroxidase activity requires the presence of H_2_O_2_ and the final outcome of the reaction (i.e., the elimination or the accumulation of ROS) depends on the type of activity cycle [39]. In addition, peroxidases are involved in a wide range of physiological processes which include cell wall metabolism, lignification, suberisation, auxin metabolism, wound healing, reactive oxygen species (ROS) production and reactive nitrogen species (RNS) metabolism, and defence against pathogens [41]_._ In our study, the up-regulation of peroxidase following SWE application strongly supports its role in controlling the levels of cellular ROS. However, the determination of H_2_O_2_ is also needed in the aerial tissues to investigate whether there is systemic accumulation in these tissues of ROS during SWE-induced priming.

Production of ROS is critical for successful activation of immune responses in plants against biotic stresses [42] and the plant NADPH oxidase, RBOHD, encoded by the *RBOHD* gene examined in our study, is a primary player in ROS production during innate immunity [43]. *A. thaliana* carries 10 genes encoding NADPH oxidases, which belong to the *RBOH* (respiratory burst oxidase homolog) family. Among them, RBOHD and, to a lesser extent, RBOHF are required for the generation of apoplastic ROS during incompatible plant–pathogen interactions. RBOHD is also required for cell death control, cell wall damage-induced lignification, and systemic signalling in response to biotic and abiotic stresses [44,45]. Another ROS-associated gene, *glutathione S-transferase* (*GST*) examined here, also functions in antioxidative reactions in order to eliminate ROS that accumulate in response to stress [46]. In our study, the induction of these two genes (*RBOHD* and *GSTF8*) in *A. thaliana* following treatment with SWE suggests their involvement in seaweed extract-induced priming. Further, our finding of the involvement of the ROS associated genes and the production of ROS in both *A. thaliana* and *S. lycopersicum* clearly demonstrates the involvement of ROS in the SWE seaweed extract-induced priming of both the model and a crop plant.

### 3.2. Effect of SWE on the Post-Challenge Primed State in A. thaliana Infected with P. cinnamomi

We also investigated the priming-related responses during a post-challenge primed state using the model of *A. thaliana* with the generalist pathogen *P. cinnamomi*. We had previously shown that a continuous application of SWE to *A. thaliana* roots suppressed *P. cinnamomi* growth through the stimulation of defence pathways [12]. To further examine these responses in our current study, we exposed *A. thaliana* to two temporally separated applications of SWE and then examined the production of ROS and the expression of major priming-related genes. Based on our experiments with one application of SWE described above, we expected, in the absence of a pathogen, the production of ROS at 3 days post-priming. We have also now shown production of ROS at 5 days post-priming following two applications of SWE. For those plants infected with *P. cinnamomi*, the production of ROS was also found in the post-challenge primed state at both 3 and 5 days, therefore demonstrating that SWE-induced priming activated ROS production during pathogen-induced stress.

For the post-challenge primed state analysed here, we have demonstrated a strong association with an up-regulation of SA-related genes. SA has a key role in plant priming against pathogens [47] and NPR1 functions as a master regulator of SA-mediated signalling pathways. Recently, the function of NPR1 as an SA receptor has been established [48]. Additionally, ROS-stimulated signals are involved both upstream and downstream in SA signalling pathways in response to stress [25]. It is also known that NPR1 interacts with TGA (TGAGG-binding) and TCP transcription factors to regulate the production of PR proteins including PR1 and PR5 [48,49]. In our study, following two SWE applications, the up-regulation of *NPR1*, the central regulator of SA signalling pathways, was found at 3 days post-priming. *PR1* and *PR5* were also found to be involved in early stages of the post-challenge primed state, whereas *MYB75* was found to contribute to the later stages. However, *NPR1* and *PR1* were expressed at the later stages of plant infection at 5 days post-priming. More importantly, all four defence priming-related genes were up-regulated at the time of inoculation in those plants harvested at 3 days after SWE application, which indicates an enhanced priming response following two applications of SWE in comparison with that of a single application. These results suggest that the variation in timing of expression for major priming-related genes is based upon the duration of the primed state following seaweed extract application. In addition, an NPR1-dependent SA-mediated signalling pathway is likely to be involved in induction of a post-challenge primed state following SWE treatment. Further, the plant defence component changes in this state suggest heightened resistance to pathogens.

### 3.3. The Mode of Action of SWE

Although the mode of action of seaweed extracts is not clear, the mechanism is not believed to be based on either their nutritional content or phytohormone composition [50] or to direct action on the pathogen [12]. Instead, a mode of action based on activating natural plant responses has emerged. Plant priming and seaweed extract biostimulants have several common attributes, their mode of action is systemic in plants, they increase tolerance to a broad range of abiotic and biotic stresses [51], they are non-specific to plant species, and they result in improved plant growth. Hence, we hypothesise that seaweed extracts stimulate, possibly through laminarins, a plant priming mechanism as part of their induction of plant responses. For example, laminarins from a range of algal species act as elicitors to induce defence in a range of plant species [6].

In the priming phase, there was induction of ROS production and the expression of key marker genes for systemic acquired resistance (SAR), and then in the primed phase there was enhanced production of ROS and key marker genes for SAR and other key priming-related genes and pathways. Furthermore, we found that the ROS response was temporal. At the post-challenge primed state we found that the magnitude of the ROS response and the up-regulation of gene expression was more pronounced. Given that plant priming is an adaptive and low-cost defence mechanism that leads to a better trade-off between growth and plant defence responses, a deeper understanding is needed if we wish to exploit this mechanism to transform agricultural food production.

## 4. Materials and Methods

### 4.1. Examination of the Priming Phase Responses in Arabidopsis thaliana and Solanum lycopersicum Following a Single Application of SWE

#### 4.1.1. *A. thaliana* Seed Germination, Growth Conditions, and Treatment with SWE

Seeds of *Arabidopsis thaliana* ecotype Ler (Lehle seeds, Round Rock, TX, USA,) were germinated and grown as previously described [12]. Briefly, sterilised seeds were placed into Petri dishes (9-cm-diameter) containing MS (Murashige and Skoog) medium supplemented with 0.8% (*w*/*v*) bacteriological agar [52]. After 14 days of growth, uniform-sized plants were transferred into sand in a growth tube. For seaweed extract treatment of seedlings of *A. thaliana,* individual seedlings were removed from the Petri dish and grown in autoclaved and sterilised commercial propagation sand (Bunnings, Waurn Ponds, Australia) that was within 5 mL plastic disposable pipette tubes (Axygen™, Pacific Laboratory Products, Blackburn, Australia) with a piece of cotton wool inserted into the narrow end to form a plug that held the sand in place. Each tube was filled to within 0.5 cm of the top and then 1 mL of distilled water was added to moisten the sand. Plants were treated with an SWE (Seasol, Bayswater, Australia) made from two brown algae, *Ascophyllum nodosum* and *Durvillaea potatorum*, using an alkaline extraction process to manufacture the SWE [3]. The SWE had 16% (*w*/*w*) soluble solids and the composition has been previously described [11]. The SWE was used at a 1 in 400 dilution for consistency with previous laboratory, greenhouse, and field studies [3,53,54,55]. One set of plants that were grown in the MS plates was carefully removed and the roots placed in SWE (1:400 in distilled water) or water as the control within a Petri dish for less than 5 s. Then, the plants were removed from the liquid and the roots dried on absorbent paper and the whole plants were frozen in liquid nitrogen. These plants were designated as the 0-day-control.

Those seedlings that were not being used as the 0-day-control were carefully removed from the MS plates and the roots were carefully placed within a 10 mm deep hole made in the sand within the tube by pushing the narrow end of another 5 mL tube into the sand. Then, a further 1 mL of distilled water was added to gently enclose the root system within the sand. Tubes containing the plants were then placed vertically within a holding rack and transferred to a plant growth chamber under the same conditions as previously described [12]. Twenty-four hours after transplantation, the seedlings were treated with SWE (700 µL of 1:400 dilution) or distilled water as the control by adding the liquid carefully and directly to the sand surface. Following treatment of plants with SWE, each day, up until 5 days after treatment, 700 µL of distilled water was added to each tube for both the SWE-treated plants and the controls (Supplementary Figure S1). On days 1, 3, and 5 after treatment with SWE, individual plants were removed from a growth tube by briefly submerging the tube into water held within a container. The tube was gently tapped to remove the plant from the tube and sand from the root system. The intact plant was then immediately placed with its roots submerged in water within a square plastic culture dish (10 cm × 10 cm) and the roots were agitated gently to remove any residual sand particles. Whole plants were then gently and briefly dried on absorbent paper and frozen in liquid nitrogen followed by storage at −80 °C.

#### 4.1.2. *Solanum lycopersicum* Growth Conditions and Treatment with SWE

Tomato seeds (*Solanum lycopersicum*, Grosse Lisse variety) were soaked for 4 h in tap water and were then surface sterilised using 1% (*v*/*v*) sodium hypochlorite for 2 min followed by a rinse with sterile distilled water for another 2 min. The sterilised seeds were then dried on absorbent paper and then placed on moistened filter paper placed in a square plastic dish (Bio-Strategy, Tullamarine, Australia). After 6 days of growth on filter paper, the seedlings were transferred into sand in the system described above for *A. thaliana*. Twenty-four hours after transfer into sand, the seedlings were treated with SWE (700 µL from 1:400 dilution) or water as the control. The seedlings were then carefully removed from the sand at 1, 3, and 5 days after treatment as described in Section 4.1.1. Final root growth data represent the mean of three biological replicates (each replicate contained 10 plants) from two independent repeats.

#### 4.1.3. Hydrogen Peroxide Detection in Roots of *A. thaliana* and *S. lycopersicum*

To detect H_2_O_2_ production in *A. thaliana* roots, roots of harvested plants were immediately transferred into a diaminobenzidine (DAB) solution (1 mg/mL dissolved in 0.01% HCl) and incubated in the dark for 3 h. The reaction was stopped by transferring the seedlings into distilled water. After staining, roots were incubated in decolourising solution (ethanol: lactic acid: glycerol = 1:1:1) at 80 °C for 20 min. The roots were then viewed under bright field microscopy (Axioskop-2 Mot Plus microscope, Zeiss, Oberkochen, Germany) and images were captured with a digital camera mounted on the microscope.

To detect H_2_O_2_ production in *S. lycopersicum,* the roots were carefully detached and then stained with DAB according to the method described by Zhou et al. [56]. Briefly, detached roots were washed with deionised water and placed in a solution containing 0.5 mg/mL of DAB (pH 5.5) and vacuum infiltrated for 1.5 min prior to incubation for 4 h at room temperature. Images of the stained roots were captured as described above for *A. thaliana*. The final images of both *A. thaliana* and *S. lycopersicum* are representative of three biological replicates (each with at least five plants) at each time point for each treatment.

#### 4.1.4. Hydrogen Peroxide and Peroxidase Quantification

Hydrogen peroxide and peroxidase were extracted from root tissues of both plant species as described by Mintoff et al. [57]. Briefly, freshly harvested roots (ten roots per replicate) were frozen in liquid N and then ground to a fine powder with a mortar and pestle and taken up into a 1.5 mL Eppendorf tube prior to adding 500 µL of 40 mM potassium phosphate buffer (pH 6.5), and then the powder was suspended by vortexing. The samples were then centrifuged for 15 min at 13,000× *g* at 4 °C. Hydrogen peroxide and peroxidase were quantified from the supernatant using a commercial kit (Amplex Red Hydrogen Peroxide/Peroxidase Assay Kit, Life Technologies, Scoresby, Australia) as per the manufacturer’s protocols, and the resulting fluorescence was measured using a microplate reader (Varioskan LUX multimode microplate reader, Thermo Scientific, Scoresby, Australia). To measure the quantity of the compounds, linear equations for H_2_O_2_ and peroxidase were generated using serial dilutions of known concentrations of H_2_O_2_ and horseradish peroxidase (HRP) and using the Amplex^TM^ Red Kit (Supplementary Figure S2). The data represent the mean of three technical replicates of each biological replicate and are expressed as µm H_2_O_2_ g^−1^ FW for hydrogen peroxide quantification and mU peroxidase g^−1^ FW for peroxidase quantification. Final quantification data represent the mean of three biological replicates.

#### 4.1.5. Gene Expression Assessment by Quantitative PCR

##### RNA Extraction and cDNA Synthesis

Total RNA was extracted from whole plants (10 plants per biological replicate) using a commercial kit (Isolate II RNA Mini Kit, Bioline, Eveleigh, Australia) following the manufacturer’s instructions. RNA concentration and integrity were determined using spectrophotometry (NanoDrop ND-1000 spectrophotometer) using the absorbance ratios of A260/280 nm and A260/230 nm. Only RNA samples with a 260/280 nm ratio between 2.0 and 2.1 proceeded to cDNA synthesis using a SensiFAST™ cDNA synthesis kit (Bioline, Eveleigh Australia) and following the manufacturer’s instructions.

##### Quantitative PCR Conditions

The primers of all tested genes were designed using Primer3Plus software (Supplementary Table S1 and the annealing temperature of each primer pair was selected using gradient qPCR. The resulting qPCR product was analysed using gel-electrophoresis to ensure the correct gene product was produced based on the primer design. Moreover, PCR efficiency of all genes was determined by a standard curve analysis using a pooled cDNA mix from all treatment conditions as template according to the method described by Taylor et al. [58]. The real time PCR amplifications were carried out using SYBR Green detection chemistry. cDNAs were run in triplicate for both target and reference genes (*Actin2* & *Actin8* for *A. thaliana* and *EF1α* [59] & *Actin7* [60] for *S. lycopersicum*) on 96-well reaction plates using the CFX Connect real-time PCR instrument (Bio-Rad, Gladesville, Australia). The reactions were performed in a total volume of 10 µL using SYBR green mix, a 1:20 dilution of cDNA template, and 0.5 µM of primers. Cycle parameters were 95 °C for 3 min and then 40 cycles at 95 °C for 10 s, 60 °C for 10 s, and 72 °C for 30 s. Expression data were normalised against two reference genes using the 2^−ΔΔCT^ method [61]. Control plants harvested at 0 day were used as the reference sample to calculate the expression level for all other time points for control and SWE treatment.

### 4.2. Examination of the Post-Challenge Primed State Responses in Arabidopsis thaliana Following Inoculation with Phytophthora cinnamomi and Two Applications of Seaweed Extract

#### 4.2.1. Plant Growth and Treatment with SWE

To further investigate the priming response during biotic stress in *A. thaliana*, the effect of more than one treatment with SWE on infection with *P. cinnamomi* was examined. In these experiments, rather than using a single application of SWE, two temporally separated applications of SWE prior to inoculation were used.

Plants were grown and were treated with seaweed extract as described above, except that SWE (700 µL, 1:400 dilution in distilled water) was added to each tube on day 2 and day 4 after transplantation. Therefore, each day, except for day 2 and day 4 after transplantation, and up until 9 days after transplantation for both the control and treated plants, 700 µL of distilled water was added to each plant growth tube (Table S2).

#### 4.2.2. Infection of *A. thaliana* with *P. cinnamomi* Zoospores

The ecotype of *A. thaliana* used here has previously been assessed as susceptible to *P. cinnamomi* [32]. Zoospores of *P. cinnamomi* were produced according to the method described by Islam et al. [62] and the zoospore density adjusted to 1×10^5^ zoospores/mL. Roots of one set of plants that had been treated twice with SWE were inoculated at seven days after transplantation by carefully dispensing, with a pipette, 700 µL of the zoospore suspension against the side wall of the plant growth tube just above the sand surface. A separate set of plants was also inoculated at nine days after transplantation. The first set of inoculated plants (eight plants/replicate/treatment) were carefully removed in groups from the sand in the growth tubes following the procedures described above on day seven immediately after inoculation and at 3, 6, 12, and 24 h post-inoculation. These plants were therefore harvested 3 days after the second application of SWE and we have designated this group as “3 days post-priming”. The second set of plants were left for a further two days, inoculated, harvested, and subjected to the same analysis as those plants harvested on day seven. These plants were designated as “5 days post-priming”. For ROS detection and gene expression analysis for these sets of plants, the procedures described above were followed.

### 4.3. Statistical Analysis

Data were analysed using International Business Machines Statistical Package for the Social Sciences (IBM SPSS) statistics and the significance of differences between or among means was obtained using Duncan’s multiple range test (DMRT) at the 0.05 level of significance.

## 5. Conclusions

Treatment of plants with the SWE derived from two brown algal species induced a typical priming response that included ROS activation and major priming-related gene expression. ROS is a clear hallmark of the priming response in both the model plant, *A. thaliana*, and a common horticultural species, *S. lycopersicum*. ROS production was closely linked to the activation of priming and defence-priming-related genes. Therefore, treatment of plants with SWE biostimulants readies them for action against potential biotic and abiotic stresses. This new role for seaweed extract-based biostimulants can now be applied to enhancing the resilience of crop species against the various challenges that compromise their productivity. Application of SWE biostimulants can also be included in the toolbox of approaches that can especially be used against plant pathogens such as those in the genus *Phytophthora*. Further studies are warranted that explore the ROS-induced upstream and downstream signalling pathways, such as those that involve SA, that may regulate plant responses to various stresses and the critical role that biostimulants can play. The elucidation of the defence pathways related to SA and SAR would also be a valuable extension to our current findings. For example, it would be important to determine the role of additional pathogen-responsive, SA-regulated, and SAR-responsive genes such as MPK3 (mitogen activated protein kinase 3), MPK6 [63], and HDAC19 (histone deacetylase) [64].

## Data Availability

Available data are provided in the publication and as supplementary information.

## Supplementary Materials

The following are available online at https://www.mdpi.com/article/10.3390/plants10112476/s1, Figure S1: Experimental outline of investigation of responses at the priming phase in *Arabidopsis thaliana* and *Solanum lycopersicum* following a single SWE application. Table S1: Primer pair sequences used in this study. Table S2: Experimental outline of investigation of responses at the post-challenge primed state in *Arabidopsis thaliana* following two SWE applications and inoculated with *Phytophthora cinnamomi* (*Pc*). Figure S2: Linear regression of various concentrations of H_2_O_2_ and horseradish peroxidase (HRP). Figure S3: Hydrogen peroxide (H_2_O_2_) detection in tomato roots grown in the sand culture system with application of SWE (1:400 dilution) or water as the control. Figure S4: The effect of seaweed extract (SWE) application on plant vigor and root growth.
