# Supplementary material for: Seaweed Extract-Stimulated Priming in Arabidopsis thaliana and Solanum lycopersicum
Source: Plants (Basel). 2021 Nov 16;10(11):2476. doi: 10.3390/plants10112476 (PMC8620570; doi:10.3390/plants10112476)
Supplement: Supplementary file 1 [file plants-10-02476-s001.zip › plants-1430670-supplementary.pdf]

Supplementary Figure S1: Experimental outline of investigation of responses at priming phase in *Arabidopsis thaliana* and *Solanum lycopersicum* following a single SWE application.

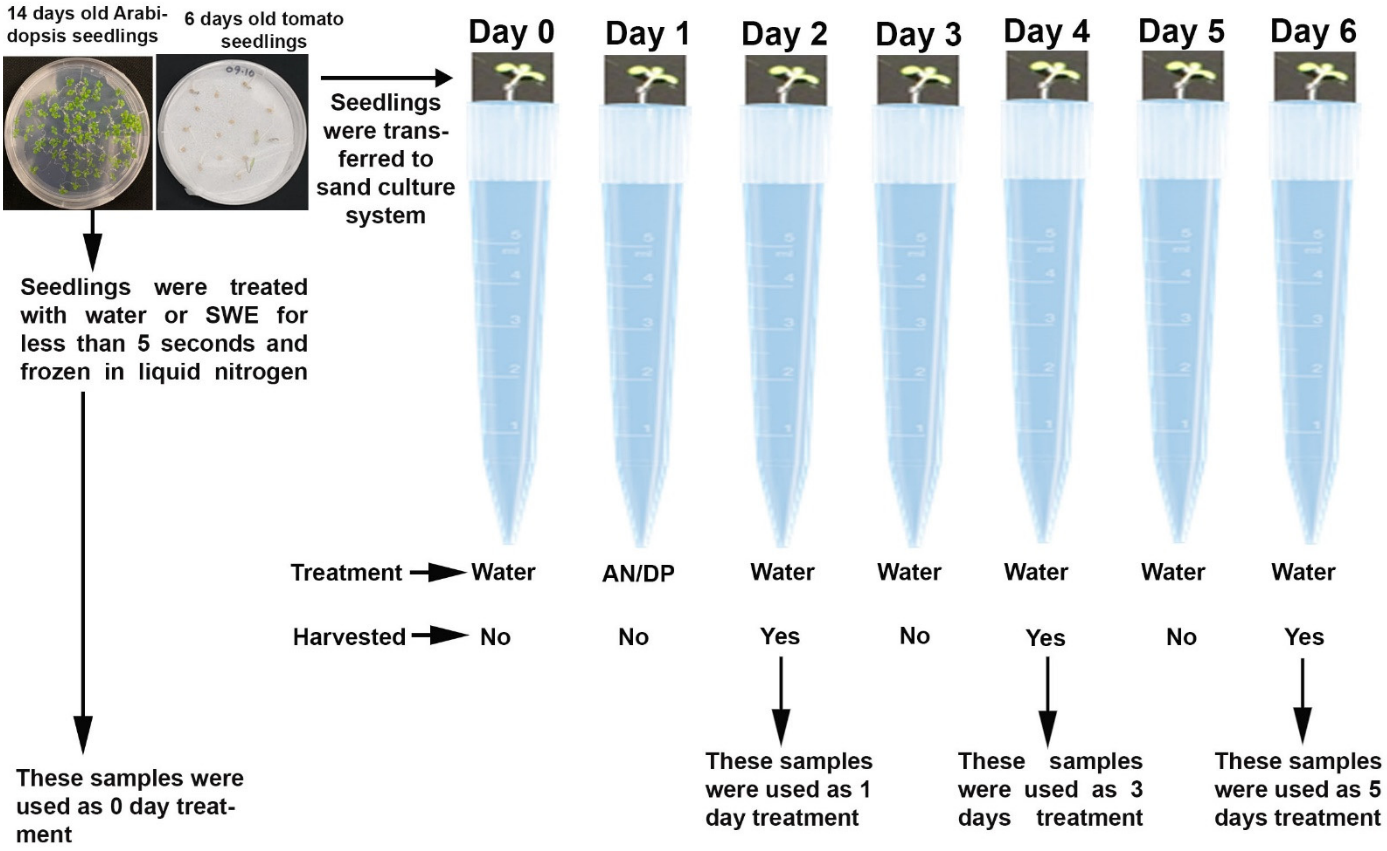

Supplementary Table S1. Primer pair sequences used in this study

Arabidopsis thaliana			
**Gene id (s)**	**Sequence**	**Product length (bp)**	**Primer source**
*ACT2*_for	ACCTTGCTGGACGTGACCTT	88	This study
*ACT2*_rev	ATTTCCCGCTCTGCTGTTGT		
*ACT8*_for	AACCAGCTCCTCCATCGAAA	130	
*ACT8*_rev	GATCCCTGCAGCTTCCATTC		
*MYB75*_for	TTCCTGCACCGGTTTAGCCC	122	
*MYB75*_rev	TTGGTGTGCATAGATTCTTCCTGA		
*PR1*_for	CGTAAGGCCCACCAGAGTGT	131	
*PR1*_rev	TCACAACCAGGCACGAGGAG		
*NPR1*_for	TACCAGTGAGACGGTCAGGC	136	
*NPR1*_rev	TGTGTCGTCCACTGTTTAGTTGC		
*PR5*_for	AGCGGCATTGCTGTTATGGC	134	
*PR5*_rev	CGGGAAGCACCTGGAGTCAA		

*PCC1*_for	GTACACAAGTCCGCCACCAA	93	
*PCC1*_rev	ACGCCCTTGGAGTTTGTCTC		
*ACD6*_for	CTTCTCGTGGTAGCGGCTCT	122	
*ACD6*_rev	GGGTTAGTGGCCAAAGTTGC		
*AED1*_for	ATTATGCTTTGCGTATGTCTCAATTGG	261	[22]
*AED1*_rev	GGACTCCACACGTGCTTGATCG		
*GRXC9*_for	TCTCAAGGACCGCCGGATTC	129	This study
*GRXC9*_rev	ACGGCGAGAGAGTTCGGATG		
*EDS16*_for	TTGGCAGGGAGACTTACGAAGG	91	
*EDS16*_rev	AATTCGAAGAAATGAAGAGCTTGGA		
*RBOHD*_for	AAGCGGGATAGTCGTCGGTG	77	
*RBOHD*_rev	CCGCGGCAATTAAACGTGGA		
*GSTF8*_for	GCAGGAGTGCGACAGAAACC	145	
*GSTF8*_rev	ACCGACAACATGAACAGTGGC		
*SAG21*_for	AGCGGTGGAAGAAGTGGAGC	140	
*SAG21*_rev	GCGTCAATCTCGTTGGAACCG		
*TPX2*_for	TGGGTCTGGAGAATACACGC	138	
*TPX2*_rev	TCGCCACCAGATTCAACATTGG		
Solanum lycopersicum			
*SlNPR1*_for	TGTGGGAAAGATAGCAGCACG	140	[31]
*SlNPR1*_rev	GTCCACACAAACACACACATC		
*SlEF1α*_for	GATTGGTGGTATTGGAACTGTC	137	[59]
*SlEF1α*_rev	AGCTTCGTGGTGCATCTC		
*SlActin7*_for	GATACCTGCAGCTTCCATACC	110	[60]
*SlActin7*_rev	GCTTTGCCGCATGCCATTCT		
*SlPR5*_for	TGGGCCGGGAAAATGTCATCC	150	This study
*SlPR5*_rev	GTAGGACCACATGGACCTTGAGTG		

Table S**2.** Experimental outline of investigation of responses at post-challenge primed state in *Arabidopsis thaliana* following 2 SWE applications and inoculated with *Phytophthora cinnamomi* (*Pc*).

**Treatment**	**Day 0**	**Day 1**	**Day 2**	**Day 3**	**Day 4**	**Day 5**	**Day 6**	**Day 7**	**Day 8**	**Day 9**
dH_2O Control	2 mL	700 µL	700 µL	700 µL	700 µL	700 µL	700 µL	**'3 days post-priming'** Inoculation with *Pc* and harvesting	700 µL	**'5 days post-priming'** Inoculation with *Pc* and harvesting
Diluted SWE	dH_2O 2 mL	dH_2O 700µL	**SWE 700 µL**	dH_2O 700µL	**SWE 700 µL**	dH_2O 700µL	dH_2O 700µL	**'3 days post-priming'** Inoculation with *Pc* and harvesting	dH_2O 700µL	**'5 days post-priming'** Inoculation with *Pc* and harvesting

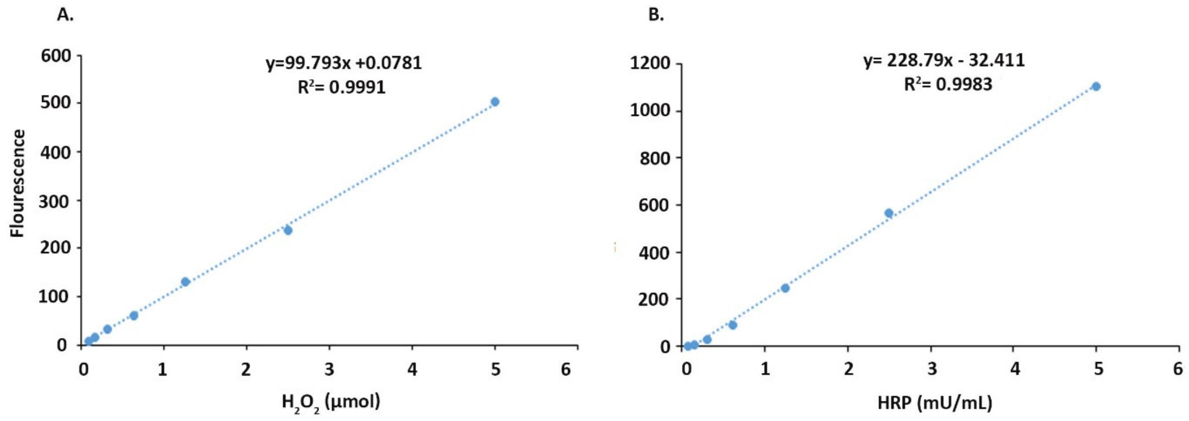

Supplementary Figure S2. Linear regression of various concentrations of H_2O_2 and horseradish peroxidase (HRP). To detect H_2O_2 and HRP, the indicated amount of H_2O_2 (A) and HRP (B) were added in reactions containing 50 µmol Amplex Red reagent and 50 mM sodium phosphate buffer, pH 7.4. Then the reactions was incubated for 30 minutes at room temperature in the dark. Fluorescent was then measured with a fluorescent microplate reader using excitation at 530 nm and fluorescent emission at 590.

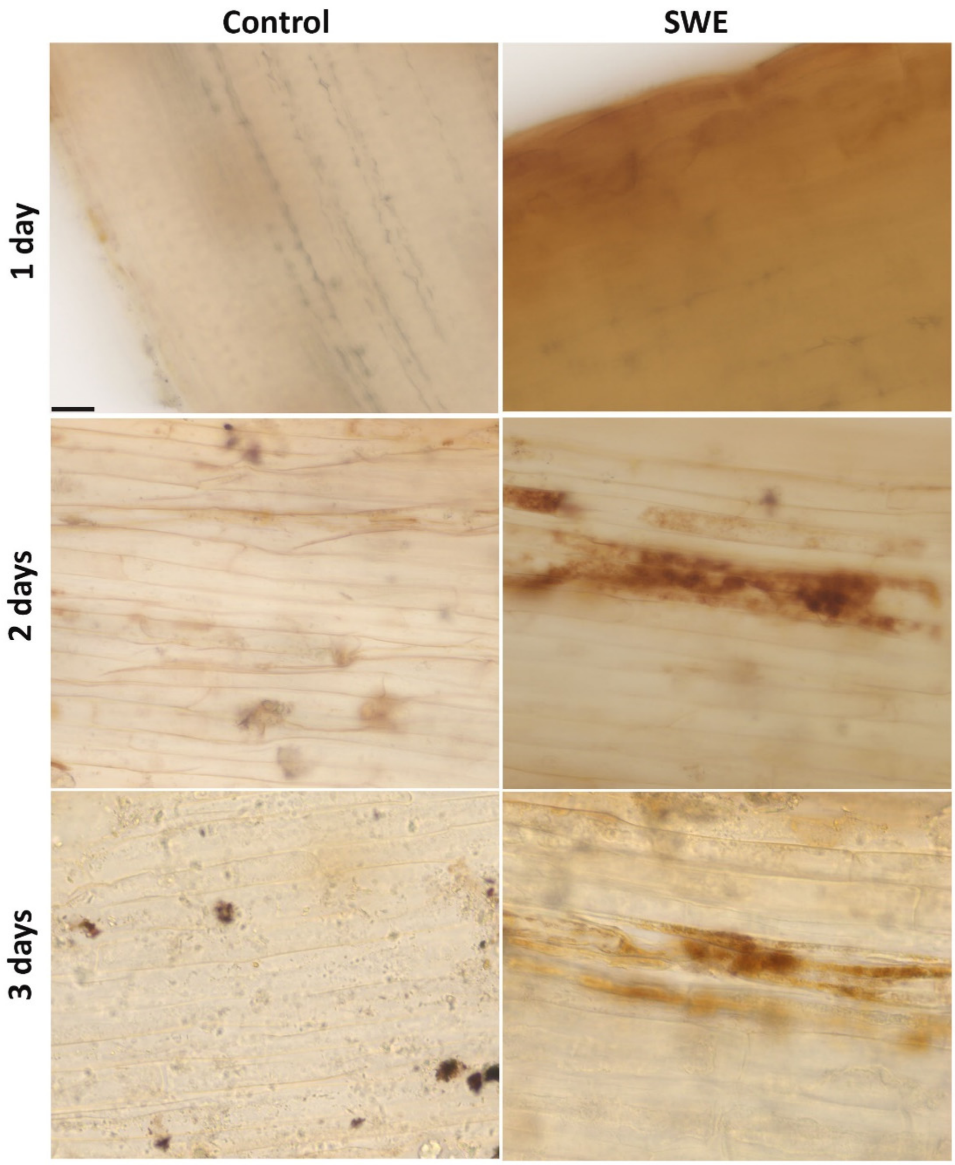

Supplementary Figure S3. Hydrogen peroxide (H_2O_2) detection in tomato roots grown in the sand culture system with application of SWE (1:400 dilution) or water as the control. Images were collected at 1, 2 and 3 days after SWE treatment. Scale bar = 20 µm. Images are representative of three independent biological replicates each with at least 12 roots.

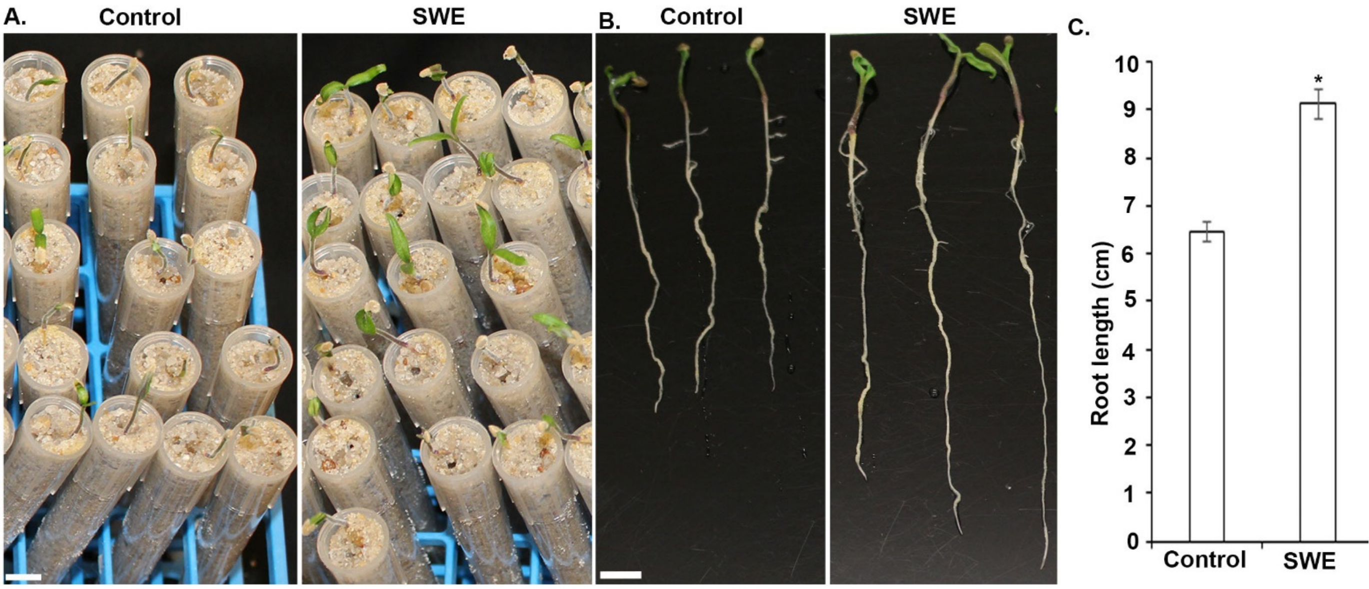

Supplementary Figure S4. The effect of seaweed extract (SWE) application on plant vigor and root growth. A. Tomato seedlings growing in sand at five days after SWE treatment or water as a control. B. Tomato plants after harvesting from sand at five days after SWE treatment or water as a control Scale bar = 1 cm C. Root length (determined using ImageJ software) for tomato seedlings at five days after treatment with SWE or water control. * denotes significant difference ($p<0.05$) between treated and control plants. Data shown are the mean of three independent biological replicates (each replicate consisted of 10 plants) and bars represent the standard error of the mean
